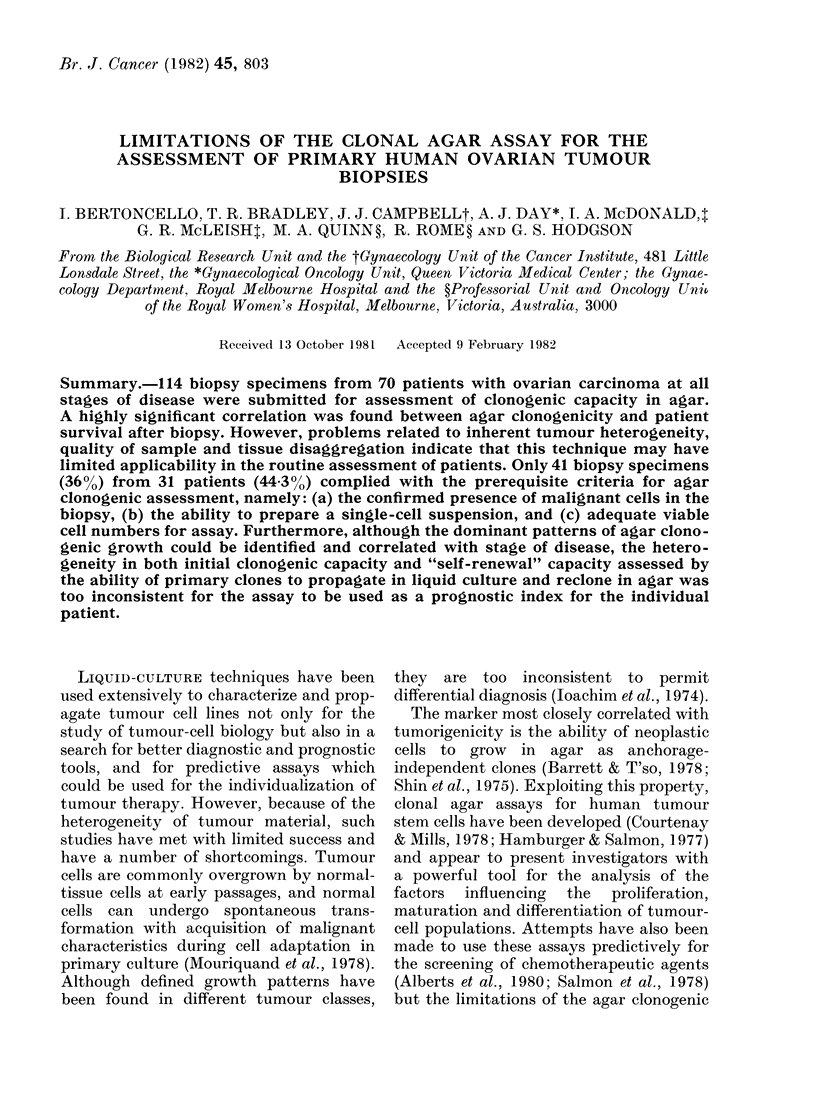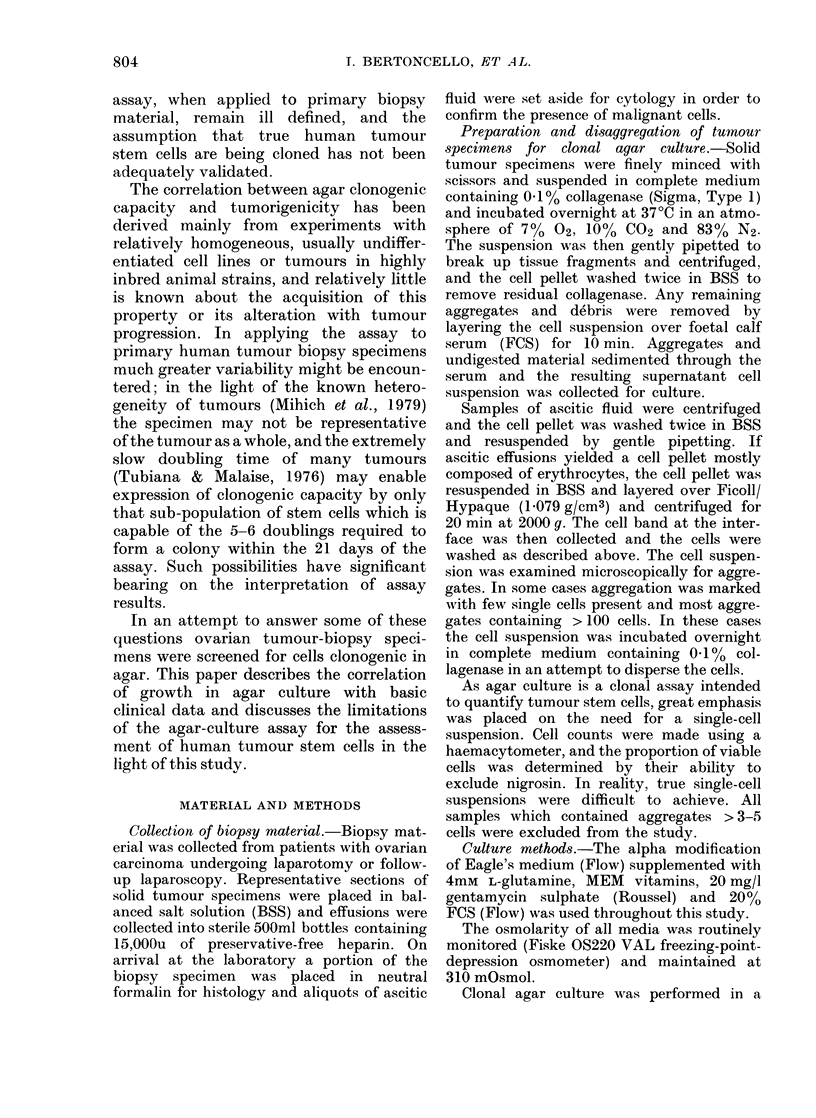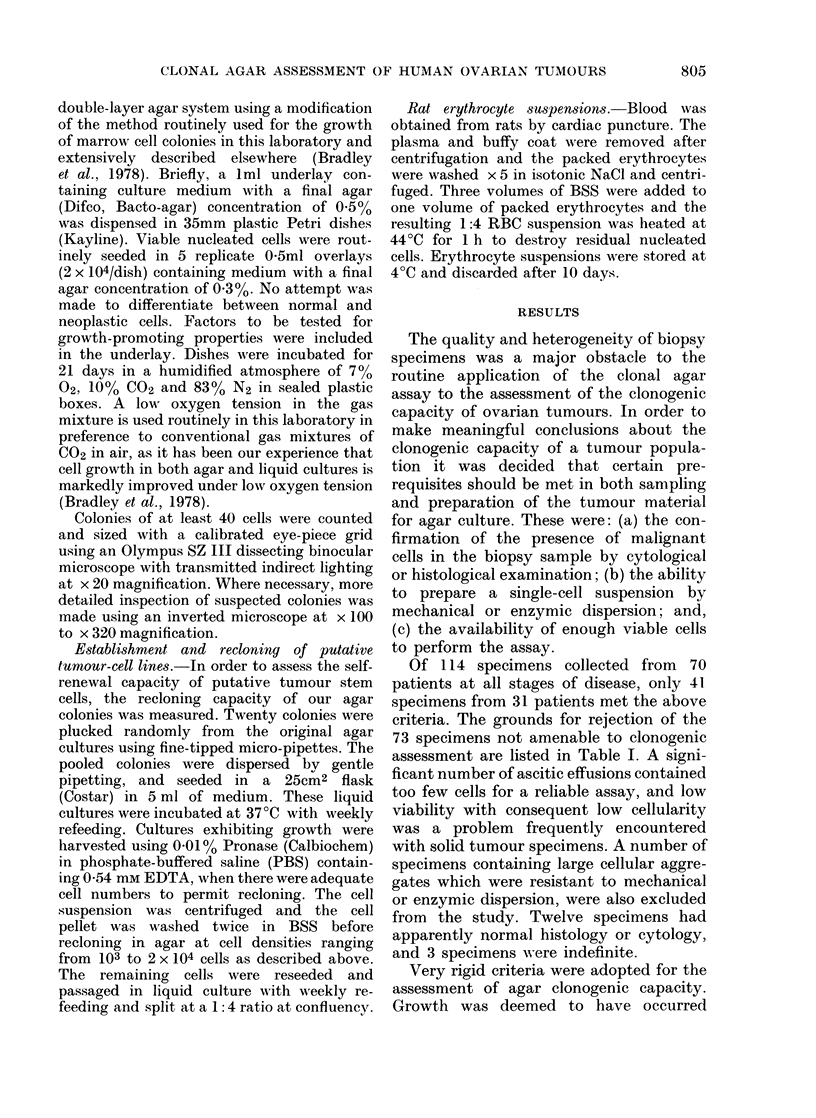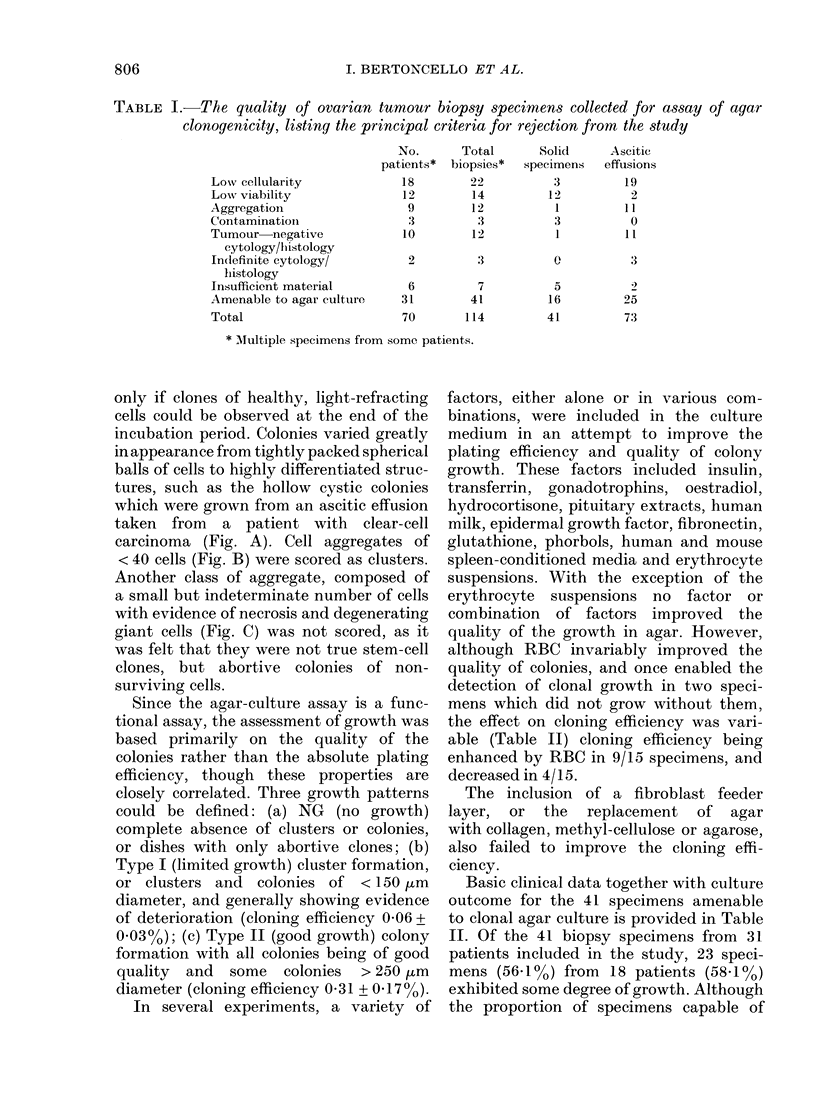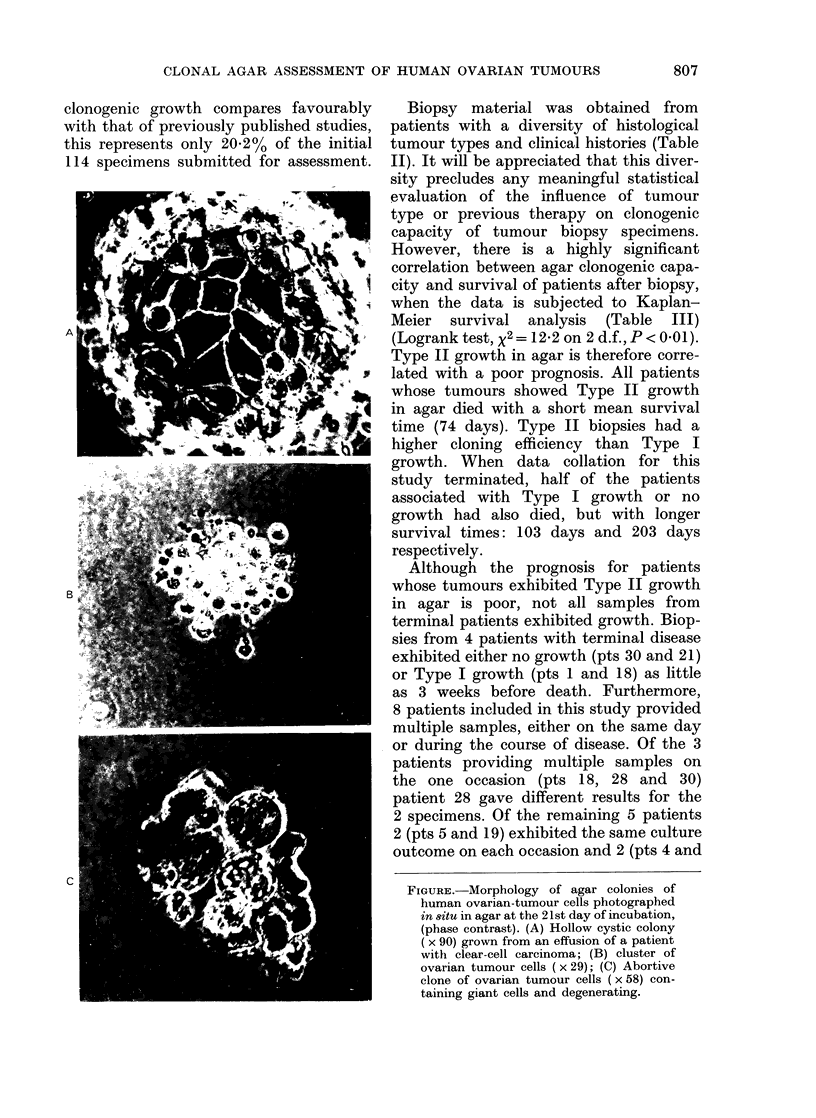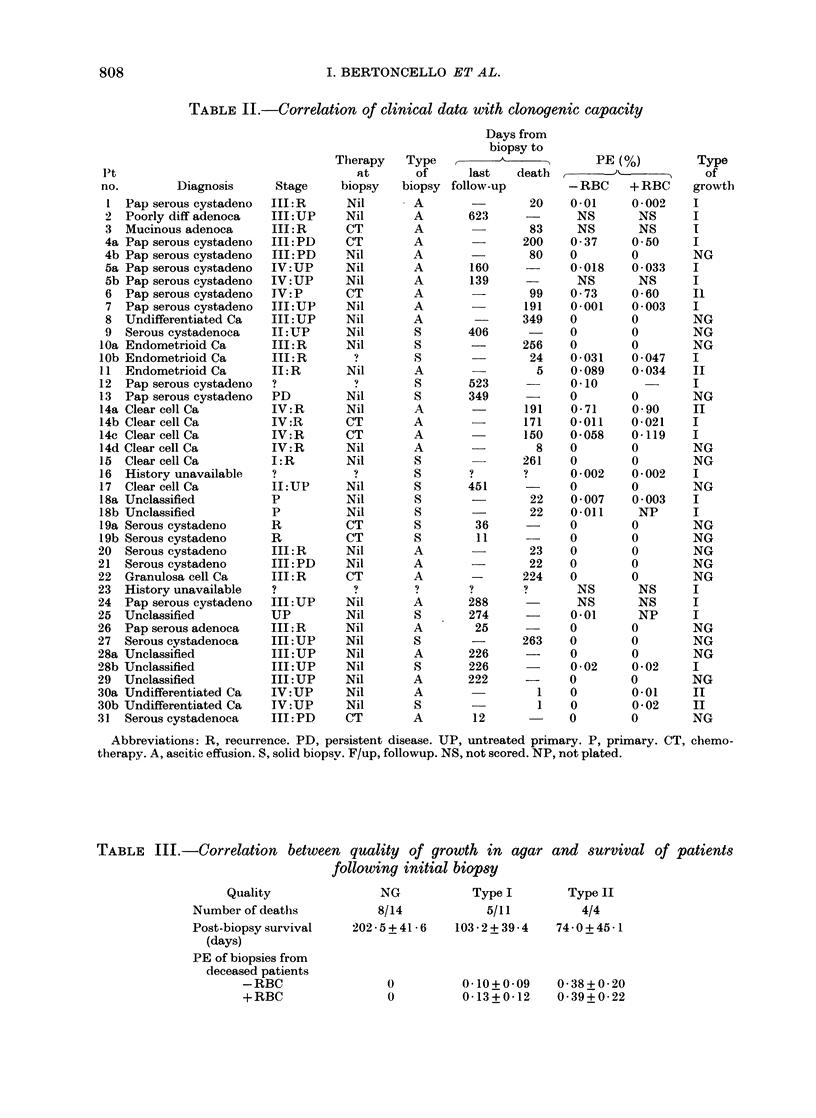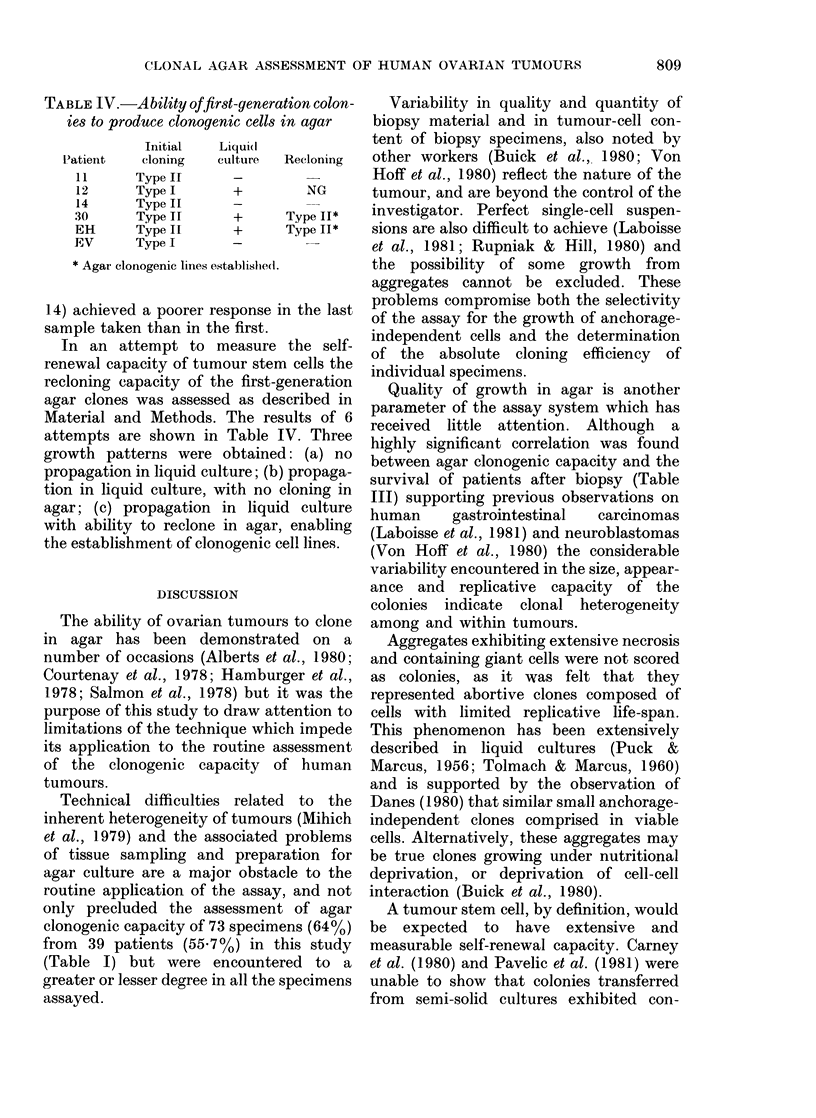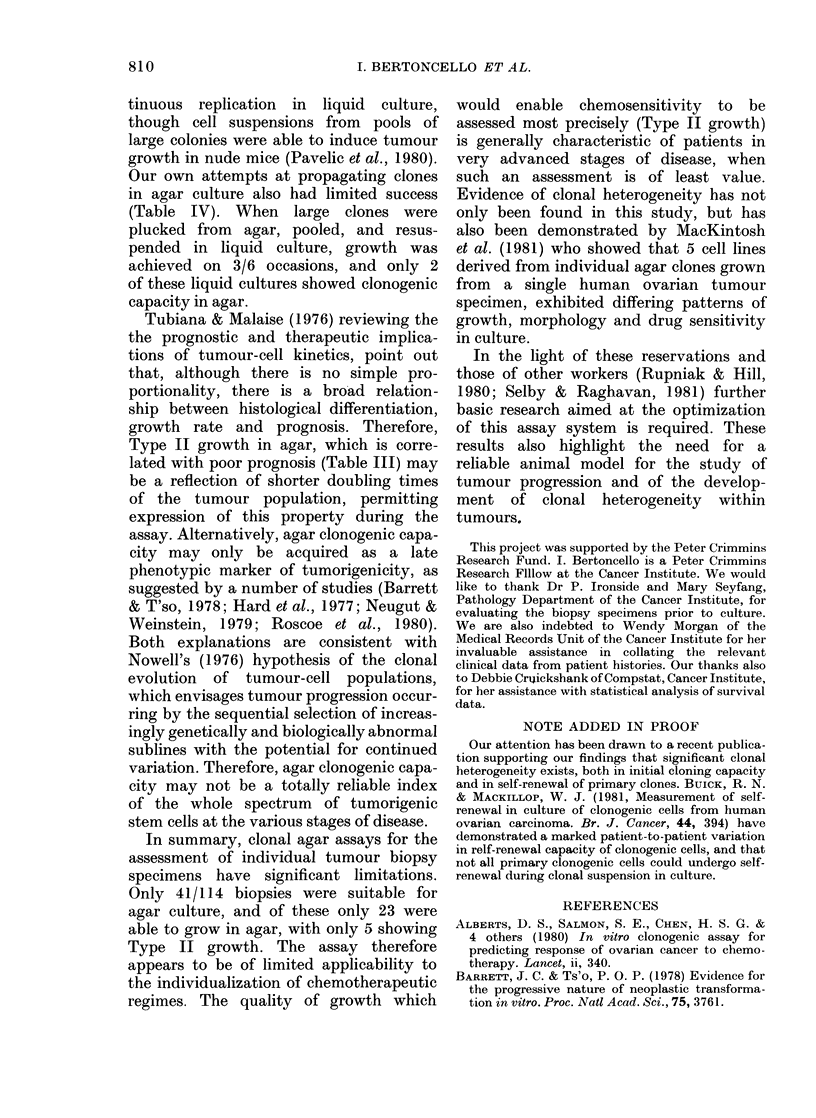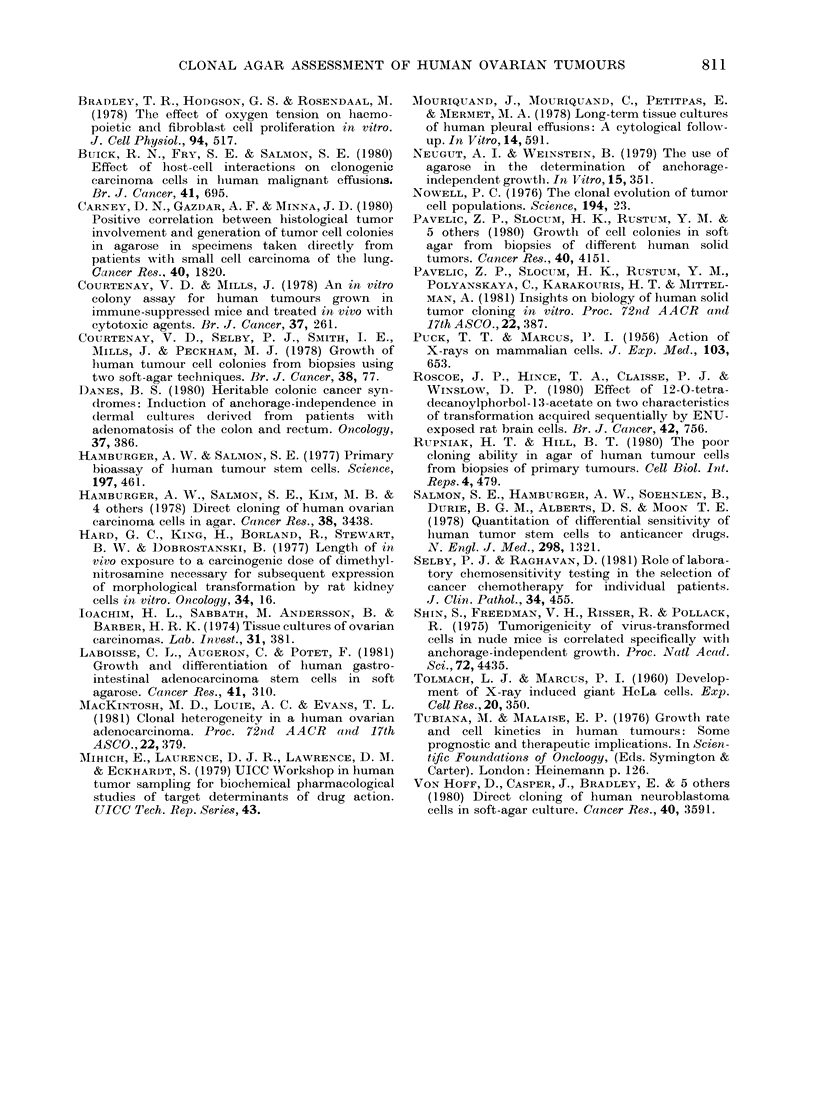# Limitations of the clonal agar assay for the assessment of primary human ovarian tumour biopsies.

**DOI:** 10.1038/bjc.1982.131

**Published:** 1982-06

**Authors:** I. Bertoncello, T. R. Bradley, J. J. Campbell, A. J. Day, I. A. McDonald, G. R. McLeish, M. A. Quinn, R. Rome, G. S. Hodgson

## Abstract

**Images:**


*Br. J. Cancer* (1982) **45**, 803

### LIMITATIONS OF THE CLONAL AGAR ASSAY FOR THE ASSESSMENT OF PRIMARY HUMAN OVARIAN TUMOUR BIOPSIES

I. BERTONCELLO, T. R. BRADLEY, J. J. CAMPBELL†, A. J. DAY*, I. A. McDONALD,‡ G. R. McLEISH‡, M. A. QUINN§, R. ROME§ AND G. S. HODGSON

*From the Biological Research Unit and the †Gynaecology Unit of the Cancer Institute,* 481 *Little Lonsdale Street, the *Gynaecological Oncology Unit, Queen Victoria Medical Center; the Gynaecology Department, Royal Melbourne Hospital and the §Professorial Unit and Oncology Unit of the Royal Women's Hospital, Melbourne, Victoria, Australia,* 3000

Received 13 October 1981 Accepted 9 February 1982

**Summary.—114 biopsy specimens from 70 patients with ovarian carcinoma at all stages of disease were submitted for assessment of clonogenic capacity in agar. A highly significant correlation was found between agar clonogenicity and patient survival after biopsy. However, problems related to inherent tumour heterogeneity, quality of sample and tissue disaggregation indicate that this technique may have limited applicability in the routine assessment of patients. Only 41 biopsy specimens (36%) from 31 patients (44·3%) complied with the prerequisite criteria for agar clonogenic assessment, namely: (a) the confirmed presence of malignant cells in the biopsy, (b) the ability to prepare a single-cell suspension, and (c) adequate viable cell numbers for assay. Furthermore, although the dominant patterns of agar clonogenic growth could be identified and correlated with stage of disease, the heterogeneity in both initial clonogenic capacity and "self-renewal" capacity assessed by the ability of primary clones to propagate in liquid culture and reclone in agar was too inconsistent for the assay to be used as a prognostic index for the individual patient.**

LIQUID-CULTURE techniques have been used extensively to characterize and propagate tumour cell lines not only for the study of tumour-cell biology but also in a search for better diagnostic and prognostic tools, and for predictive assays which could be used for the individualization of tumour therapy. However, because of the heterogeneity of tumour material, such studies have met with limited success and have a number of shortcomings. Tumour cells are commonly overgrown by normal-tissue cells at early passages, and normal cells can undergo spontaneous transformation with acquisition of malignant characteristics during cell adaptation in primary culture (Mouriquand *et al.*, 1978). Although defined growth patterns have been found in different tumour classes, they are too inconsistent to permit differential diagnosis (Ioachim *et al.*, 1974).

The marker most closely correlated with tumorigenicity is the ability of neoplastic cells to grow in agar as anchorage-independent clones (Barrett & T'so, 1978; Shin *et al.*, 1975). Exploiting this property, clonal agar assays for human tumour stem cells have been developed (Courtenay & Mills, 1978; Hamburger & Salmon, 1977) and appear to present investigators with a powerful tool for the analysis of the factors influencing the proliferation, maturation and differentiation of tumour-cell populations. Attempts have also been made to use these assays predictively for the screening of chemotherapeutic agents (Alberts *et al.*, 1980; Salmon *et al.*, 1978) but the limitations of the agar clonogenic assay, when applied to primary biopsy material, remain ill defined, and the assumption that true human tumour stem cells are being cloned has not been adequately validated.

The correlation between agar clonogenic capacity and tumorigenicity has been derived mainly from experiments with relatively homogeneous, usually undifferentiated cell lines or tumours in highly inbred animal strains, and relatively little is known about the acquisition of this property or its alteration with tumour progression. In applying the assay to primary human tumour biopsy specimens much greater variability might be encountered; in the light of the known heterogeneity of tumours (Mihich *et al.*, 1979) the specimen may not be representative of the tumour as a whole, and the extremely slow doubling time of many tumours (Tubiana & Malaise, 1976) may enable expression of clonogenic capacity by only that sub-population of stem cells which is capable of the 5–6 doublings required to form a colony within the 21 days of the assay. Such possibilities have significant bearing on the interpretation of assay results.

In an attempt to answer some of these questions ovarian tumour-biopsy specimens were screened for cells clonogenic in agar. This paper describes the correlation of growth in agar culture with basic clinical data and discusses the limitations of the agar-culture assay for the assessment of human tumour stem cells in the light of this study.

### MATERIAL AND METHODS

*Collection of biopsy material.*—Biopsy material was collected from patients with ovarian carcinoma undergoing laparotomy or follow-up laparoscopy. Representative sections of solid tumour specimens were placed in balanced salt solution (BSS) and effusions were collected into sterile 500ml bottles containing 15,000u of preservative-free heparin. On arrival at the laboratory a portion of the biopsy specimen was placed in neutral formalin for histology and aliquots of ascitic fluid were set aside for cytology in order to confirm the presence of malignant cells.

*Preparation and disaggregation of tumour specimens for clonal agar culture.*—Solid tumour specimens were finely minced with scissors and suspended in complete medium containing 0·1% collagenase (Sigma, Type 1) and incubated overnight at 37°C in an atmosphere of 7% $O_2$, 10% $CO_2$ and 83% $N_2$. The suspension was then gently pipetted to break up tissue fragments and centrifuged, and the cell pellet washed twice in BSS to remove residual collagenase. Any remaining aggregates and débris were removed by layering the cell suspension over foetal calf serum (FCS) for 10 min. Aggregates and undigested material sedimented through the serum and the resulting supernatant cell suspension was collected for culture.

Samples of ascitic fluid were centrifuged and the cell pellet was washed twice in BSS and resuspended by gentle pipetting. If ascitic effusions yielded a cell pellet mostly composed of erythrocytes, the cell pellet was resuspended in BSS and layered over Ficoll/Hypaque (1·079 g/cm$^3$) and centrifuged for 20 min at 2000 *g*. The cell band at the interface was then collected and the cells were washed as described above. The cell suspension was examined microscopically for aggregates. In some cases aggregation was marked with few single cells present and most aggregates containing $>100$ cells. In these cases the cell suspension was incubated overnight in complete medium containing 0·1% collagenase in an attempt to disperse the cells.

As agar culture is a clonal assay intended to quantify tumour stem cells, great emphasis was placed on the need for a single-cell suspension. Cell counts were made using a haemacytometer, and the proportion of viable cells was determined by their ability to exclude nigrosin. In reality, true single-cell suspensions were difficult to achieve. All samples which contained aggregates $>3$–5 cells were excluded from the study.

*Culture methods.*—The alpha modification of Eagle's medium (Flow) supplemented with 4mM L-glutamine, MEM vitamins, 20 mg/l gentamycin sulphate (Roussel) and 20% FCS (Flow) was used throughout this study.

The osmolarity of all media was routinely monitored (Fiske OS220 VAL freezing-point-depression osmometer) and maintained at 310 mOsmol.

Clonal agar culture was performed in a double-layer agar system using a modification of the method routinely used for the growth of marrow cell colonies in this laboratory and extensively described elsewhere (Bradley *et al.*, 1978). Briefly, a 1ml underlay containing culture medium with a final agar (Difco, Bacto-agar) concentration of 0·5% was dispensed in 35mm plastic Petri dishes (Kayline). Viable nucleated cells were routinely seeded in 5 replicate 0·5ml overlays ($2 \times 10^4$/dish) containing medium with a final agar concentration of 0·3%. No attempt was made to differentiate between normal and neoplastic cells. Factors to be tested for growth-promoting properties were included in the underlay. Dishes were incubated for 21 days in a humidified atmosphere of 7% $O_2$, 10% $CO_2$ and 83% $N_2$ in sealed plastic boxes. A low oxygen tension in the gas mixture is used routinely in this laboratory in preference to conventional gas mixtures of $CO_2$ in air, as it has been our experience that cell growth in both agar and liquid cultures is markedly improved under low oxygen tension (Bradley *et al.*, 1978).

Colonies of at least 40 cells were counted and sized with a calibrated eye-piece grid using an Olympus SZ III dissecting binocular microscope with transmitted indirect lighting at ×20 magnification. Where necessary, more detailed inspection of suspected colonies was made using an inverted microscope at ×100 to ×320 magnification.

*Establishment and recloning of putative tumour-cell lines.*—In order to assess the self-renewal capacity of putative tumour stem cells, the recloning capacity of our agar colonies was measured. Twenty colonies were plucked randomly from the original agar cultures using fine-tipped micro-pipettes. The pooled colonies were dispersed by gentle pipetting, and seeded in a 25cm² flask (Costar) in 5 ml of medium. These liquid cultures were incubated at 37°C with weekly refeeding. Cultures exhibiting growth were harvested using 0·01% Pronase (Calbiochem) in phosphate-buffered saline (PBS) containing 0·54 mM EDTA, when there were adequate cell numbers to permit recloning. The cell suspension was centrifuged and the cell pellet was washed twice in BSS before recloning in agar at cell densities ranging from $10^3$ to $2 \times 10^4$ cells as described above. The remaining cells were reseeded and passaged in liquid culture with weekly re-feeding and split at a 1:4 ratio at confluency.

*Rat erythrocyte suspensions.*—Blood was obtained from rats by cardiac puncture. The plasma and buffy coat were removed after centrifugation and the packed erythrocytes were washed ×5 in isotonic NaCl and centrifuged. Three volumes of BSS were added to one volume of packed erythrocytes and the resulting 1:4 RBC suspension was heated at 44°C for 1 h to destroy residual nucleated cells. Erythrocyte suspensions were stored at 4°C and discarded after 10 days.

### RESULTS

The quality and heterogeneity of biopsy specimens was a major obstacle to the routine application of the clonal agar assay to the assessment of the clonogenic capacity of ovarian tumours. In order to make meaningful conclusions about the clonogenic capacity of a tumour population it was decided that certain prerequisites should be met in both sampling and preparation of the tumour material for agar culture. These were: (a) the confirmation of the presence of malignant cells in the biopsy sample by cytological or histological examination; (b) the ability to prepare a single-cell suspension by mechanical or enzymic dispersion; and, (c) the availability of enough viable cells to perform the assay.

Of 114 specimens collected from 70 patients at all stages of disease, only 41 specimens from 31 patients met the above criteria. The grounds for rejection of the 73 specimens not amenable to clonogenic assessment are listed in Table I. A significant number of ascitic effusions contained too few cells for a reliable assay, and low viability with consequent low cellularity was a problem frequently encountered with solid tumour specimens. A number of specimens containing large cellular aggregates which were resistant to mechanical or enzymic dispersion, were also excluded from the study. Twelve specimens had apparently normal histology or cytology, and 3 specimens were indefinite.

Very rigid criteria were adopted for the assessment of agar clonogenic capacity. Growth was deemed to have occurred

TABLE I.—*The quality of ovarian tumour biopsy specimens collected for assay of agar clonogenicity, listing the principal criteria for rejection from the study*

| | No. patients* | Total biopsies* | Solid specimens | Ascitic effusions |
|---|---|---|---|---|
| Low cellularity | 18 | 22 | 3 | 19 |
| Low viability | 12 | 14 | 12 | 2 |
| Aggregation | 9 | 12 | 1 | 11 |
| Contamination | 3 | 3 | 3 | 0 |
| Tumour—negative cytology/histology | 10 | 12 | 1 | 11 |
| Indefinite cytology/ histology | 2 | 3 | 0 | 3 |
| Insufficient material | 6 | 7 | 5 | 2 |
| Amenable to agar culture | 31 | 41 | 16 | 25 |
| Total | 70 | 114 | 41 | 73 |

* Multiple specimens from some patients.

only if clones of healthy, light-refracting cells could be observed at the end of the incubation period. Colonies varied greatly in appearance from tightly packed spherical balls of cells to highly differentiated structures, such as the hollow cystic colonies which were grown from an ascitic effusion taken from a patient with clear-cell carcinoma (Fig. A). Cell aggregates of $< 40$ cells (Fig. B) were scored as clusters. Another class of aggregate, composed of a small but indeterminate number of cells with evidence of necrosis and degenerating giant cells (Fig. C) was not scored, as it was felt that they were not true stem-cell clones, but abortive colonies of non-surviving cells.

Since the agar-culture assay is a functional assay, the assessment of growth was based primarily on the quality of the colonies rather than the absolute plating efficiency, though these properties are closely correlated. Three growth patterns could be defined: (a) NG (no growth) complete absence of clusters or colonies, or dishes with only abortive clones; (b) Type I (limited growth) cluster formation, or clusters and colonies of $< 150\ \mu m$ diameter, and generally showing evidence of deterioration (cloning efficiency $0{\cdot}06 \pm 0{\cdot}03\%$); (c) Type II (good growth) colony formation with all colonies being of good quality and some colonies $> 250\ \mu m$ diameter (cloning efficiency $0{\cdot}31 \pm 0{\cdot}17\%$).

In several experiments, a variety of factors, either alone or in various combinations, were included in the culture medium in an attempt to improve the plating efficiency and quality of colony growth. These factors included insulin, transferrin, gonadotrophins, oestradiol, hydrocortisone, pituitary extracts, human milk, epidermal growth factor, fibronectin, glutathione, phorbols, human and mouse spleen-conditioned media and erythrocyte suspensions. With the exception of the erythrocyte suspensions no factor or combination of factors improved the quality of the growth in agar. However, although RBC invariably improved the quality of colonies, and once enabled the detection of clonal growth in two specimens which did not grow without them, the effect on cloning efficiency was variable (Table II) cloning efficiency being enhanced by RBC in 9/15 specimens, and decreased in 4/15.

The inclusion of a fibroblast feeder layer, or the replacement of agar with collagen, methyl-cellulose or agarose, also failed to improve the cloning efficiency.

Basic clinical data together with culture outcome for the 41 specimens amenable to clonal agar culture is provided in Table II. Of the 41 biopsy specimens from 31 patients included in the study, 23 specimens (56·1%) from 18 patients (58·1%) exhibited some degree of growth. Although the proportion of specimens capable of clonogenic growth compares favourably with that of previously published studies, this represents only 20·2% of the initial 114 specimens submitted for assessment.

Biopsy material was obtained from patients with a diversity of histological tumour types and clinical histories (Table II). It will be appreciated that this diversity precludes any meaningful statistical evaluation of the influence of tumour type or previous therapy on clonogenic capacity of tumour biopsy specimens. However, there is a highly significant correlation between agar clonogenic capacity and survival of patients after biopsy, when the data is subjected to Kaplan–Meier survival analysis (Table III) (Logrank test, $\chi^2 = 12{\cdot}2$ on 2 d.f., $P < 0{\cdot}01$). Type II growth in agar is therefore correlated with a poor prognosis. All patients whose tumours showed Type II growth in agar died with a short mean survival time (74 days). Type II biopsies had a higher cloning efficiency than Type I growth. When data collation for this study terminated, half of the patients associated with Type I growth or no growth had also died, but with longer survival times: 103 days and 203 days respectively.

Although the prognosis for patients whose tumours exhibited Type II growth in agar is poor, not all samples from terminal patients exhibited growth. Biopsies from 4 patients with terminal disease exhibited either no growth (pts 30 and 21) or Type I growth (pts 1 and 18) as little as 3 weeks before death. Furthermore, 8 patients included in this study provided multiple samples, either on the same day or during the course of disease. Of the 3 patients providing multiple samples on the one occasion (pts 18, 28 and 30) patient 28 gave different results for the 2 specimens. Of the remaining 5 patients 2 (pts 5 and 19) exhibited the same culture outcome on each occasion and 2 (pts 4 and

FIGURE.—Morphology of agar colonies of human ovarian-tumour cells photographed *in situ* in agar at the 21st day of incubation, (phase contrast). (A) Hollow cystic colony (×90) grown from an effusion of a patient with clear-cell carcinoma; (B) cluster of ovarian tumour cells (×29); (C) Abortive clone of ovarian tumour cells (×58) containing giant cells and degenerating.

TABLE II.—*Correlation of clinical data with clonogenic capacity*

| Pt no. | Diagnosis | Stage | Therapy at biopsy | Type of biopsy | Days from biopsy to last follow-up | Days from biopsy to death | PE (%) −RBC | PE (%) +RBC | Type of growth |
|---|---|---|---|---|---|---|---|---|---|
| 1 | Pap serous cystadeno | III:R | Nil | A | — | 20 | 0·01 | 0·002 | I |
| 2 | Poorly diff adenoca | III:UP | Nil | A | 623 | — | NS | NS | I |
| 3 | Mucinous adenoca | III:R | CT | A | — | 83 | NS | NS | I |
| 4a | Pap serous cystadeno | III:PD | CT | A | — | 200 | 0·37 | 0·50 | I |
| 4b | Pap serous cystadeno | III:PD | Nil | A | — | 80 | 0 | 0 | NG |
| 5a | Pap serous cystadeno | IV:UP | Nil | A | 160 | — | 0·018 | 0·033 | I |
| 5b | Pap serous cystadeno | IV:UP | Nil | A | 139 | — | NS | NS | I |
| 6 | Pap serous cystadeno | IV:P | CT | A | — | 99 | 0·73 | 0·60 | II |
| 7 | Pap serous cystadeno | III:UP | Nil | A | — | 191 | 0·001 | 0·003 | I |
| 8 | Undifferentiated Ca | III:UP | Nil | A | — | 349 | 0 | 0 | NG |
| 9 | Serous cystadenoca | II:UP | Nil | S | 406 | — | 0 | 0 | NG |
| 10a | Endometrioid Ca | III:R | Nil | S | — | 256 | 0 | 0 | NG |
| 10b | Endometrioid Ca | III:R | ? | S | — | 24 | 0·031 | 0·047 | I |
| 11 | Endometrioid Ca | II:R | Nil | A | — | 5 | 0·089 | 0·034 | II |
| 12 | Pap serous cystadeno | ? | ? | S | 523 | — | 0·10 | — | I |
| 13 | Pap serous cystadeno | PD | Nil | S | 349 | — | 0 | 0 | NG |
| 14a | Clear cell Ca | IV:R | Nil | A | — | 191 | 0·71 | 0·90 | II |
| 14b | Clear cell Ca | IV:R | CT | A | — | 171 | 0·011 | 0·021 | I |
| 14c | Clear cell Ca | IV:R | CT | A | — | 150 | 0·058 | 0·119 | I |
| 14d | Clear cell Ca | IV:R | Nil | A | — | 8 | 0 | 0 | NG |
| 15 | Clear cell Ca | I:R | Nil | S | — | 261 | 0 | 0 | NG |
| 16 | History unavailable | ? | ? | S | ? | ? | 0·002 | 0·002 | I |
| 17 | Clear cell Ca | II:UP | Nil | S | 451 | — | 0 | 0 | NG |
| 18a | Unclassified | P | Nil | S | — | 22 | 0·007 | 0·003 | I |
| 18b | Unclassified | P | Nil | S | — | 22 | 0·011 | NP | I |
| 19a | Serous cystadeno | R | CT | S | 36 | — | 0 | 0 | NG |
| 19b | Serous cystadeno | R | CT | S | 11 | — | 0 | 0 | NG |
| 20 | Serous cystadeno | III:R | Nil | A | — | 23 | 0 | 0 | NG |
| 21 | Serous cystadeno | III:PD | Nil | A | — | 22 | 0 | 0 | NG |
| 22 | Granulosa cell Ca | III:R | CT | A | — | 224 | 0 | 0 | NG |
| 23 | History unavailable | ? | ? | ? | ? | ? | NS | NS | I |
| 24 | Pap serous cystadeno | III:UP | Nil | A | 288 | — | NS | NS | I |
| 25 | Unclassified | UP | Nil | S | 274 | — | 0·01 | NP | I |
| 26 | Pap serous adenoca | III:R | Nil | A | 25 | — | 0 | 0 | NG |
| 27 | Serous cystadenoca | III:UP | Nil | S | — | 263 | 0 | 0 | NG |
| 28a | Unclassified | III:UP | Nil | A | 226 | — | 0 | 0 | NG |
| 28b | Unclassified | III:UP | Nil | S | 226 | — | 0·02 | 0·02 | I |
| 29 | Unclassified | III:UP | Nil | A | 222 | — | 0 | 0 | NG |
| 30a | Undifferentiated Ca | IV:UP | Nil | A | — | 1 | 0 | 0·01 | II |
| 30b | Undifferentiated Ca | IV:UP | Nil | S | — | 1 | 0 | 0·02 | II |
| 31 | Serous cystadenoca | III:PD | CT | A | 12 | — | 0 | 0 | NG |

Abbreviations: R, recurrence. PD, persistent disease. UP, untreated primary. P, primary. CT, chemotherapy. A, ascitic effusion. S, solid biopsy. F/up, followup. NS, not scored. NP, not plated.

TABLE III.—*Correlation between quality of growth in agar and survival of patients following initial biopsy*

| Quality | NG | Type I | Type II |
|---|---|---|---|
| Number of deaths | 8/14 | 5/11 | 4/4 |
| Post-biopsy survival (days) | 202·5±41·6 | 103·2±39·4 | 74·0±45·1 |
| PE of biopsies from deceased patients | | | |
| −RBC | 0 | 0·10±0·09 | 0·38±0·20 |
| +RBC | 0 | 0·13±0·12 | 0·39±0·22 |

TABLE IV.—*Ability of first-generation colonies to produce clonogenic cells in agar*

| Patient | Initial cloning | Liquid culture | Recloning |
|---|---|---|---|
| 11 | Type II | − | — |
| 12 | Type I | + | NG |
| 14 | Type II | − | — |
| 30 | Type II | + | Type II* |
| EH | Type II | + | Type II* |
| EV | Type I | − | — |

* Agar clonogenic lines established.

14) achieved a poorer response in the last sample taken than in the first.

In an attempt to measure the self-renewal capacity of tumour stem cells the recloning capacity of the first-generation agar clones was assessed as described in Material and Methods. The results of 6 attempts are shown in Table IV. Three growth patterns were obtained: (a) no propagation in liquid culture; (b) propagation in liquid culture, with no cloning in agar; (c) propagation in liquid culture with ability to reclone in agar, enabling the establishment of clonogenic cell lines.

### DISCUSSION

The ability of ovarian tumours to clone in agar has been demonstrated on a number of occasions (Alberts *et al.*, 1980; Courtenay *et al.*, 1978; Hamburger *et al.*, 1978; Salmon *et al.*, 1978) but it was the purpose of this study to draw attention to limitations of the technique which impede its application to the routine assessment of the clonogenic capacity of human tumours.

Technical difficulties related to the inherent heterogeneity of tumours (Mihich *et al.*, 1979) and the associated problems of tissue sampling and preparation for agar culture are a major obstacle to the routine application of the assay, and not only precluded the assessment of agar clonogenic capacity of 73 specimens (64%) from 39 patients (55·7%) in this study (Table I) but were encountered to a greater or lesser degree in all the specimens assayed.

Variability in quality and quantity of biopsy material and in tumour-cell content of biopsy specimens, also noted by other workers (Buick *et al.*, 1980; Von Hoff *et al.*, 1980) reflect the nature of the tumour, and are beyond the control of the investigator. Perfect single-cell suspensions are also difficult to achieve (Laboisse *et al.*, 1981; Rupniak & Hill, 1980) and the possibility of some growth from aggregates cannot be excluded. These problems compromise both the selectivity of the assay for the growth of anchorage-independent cells and the determination of the absolute cloning efficiency of individual specimens.

Quality of growth in agar is another parameter of the assay system which has received little attention. Although a highly significant correlation was found between agar clonogenic capacity and the survival of patients after biopsy (Table III) supporting previous observations on human gastrointestinal carcinomas (Laboisse *et al.*, 1981) and neuroblastomas (Von Hoff *et al.*, 1980) the considerable variability encountered in the size, appearance and replicative capacity of the colonies indicate clonal heterogeneity among and within tumours.

Aggregates exhibiting extensive necrosis and containing giant cells were not scored as colonies, as it was felt that they represented abortive clones composed of cells with limited replicative life-span. This phenomenon has been extensively described in liquid cultures (Puck & Marcus, 1956; Tolmach & Marcus, 1960) and is supported by the observation of Danes (1980) that similar small anchorage-independent clones comprised in viable cells. Alternatively, these aggregates may be true clones growing under nutritional deprivation, or deprivation of cell-cell interaction (Buick *et al.*, 1980).

A tumour stem cell, by definition, would be expected to have extensive and measurable self-renewal capacity. Carney *et al.* (1980) and Pavelic *et al.* (1981) were unable to show that colonies transferred from semi-solid cultures exhibited continuous replication in liquid culture, though cell suspensions from pools of large colonies were able to induce tumour growth in nude mice (Pavelic *et al.*, 1980). Our own attempts at propagating clones in agar culture also had limited success (Table IV). When large clones were plucked from agar, pooled, and resuspended in liquid culture, growth was achieved on 3/6 occasions, and only 2 of these liquid cultures showed clonogenic capacity in agar.

Tubiana & Malaise (1976) reviewing the the prognostic and therapeutic implications of tumour-cell kinetics, point out that, although there is no simple proportionality, there is a broad relationship between histological differentiation, growth rate and prognosis. Therefore, Type II growth in agar, which is correlated with poor prognosis (Table III) may be a reflection of shorter doubling times of the tumour population, permitting expression of this property during the assay. Alternatively, agar clonogenic capacity may only be acquired as a late phenotypic marker of tumorigenicity, as suggested by a number of studies (Barrett & T'so, 1978; Hard *et al.*, 1977; Neugut & Weinstein, 1979; Roscoe *et al.*, 1980). Both explanations are consistent with Nowell's (1976) hypothesis of the clonal evolution of tumour-cell populations, which envisages tumour progression occurring by the sequential selection of increasingly genetically and biologically abnormal sublines with the potential for continued variation. Therefore, agar clonogenic capacity may not be a totally reliable index of the whole spectrum of tumorigenic stem cells at the various stages of disease.

In summary, clonal agar assays for the assessment of individual tumour biopsy specimens have significant limitations. Only 41/114 biopsies were suitable for agar culture, and of these only 23 were able to grow in agar, with only 5 showing Type II growth. The assay therefore appears to be of limited applicability to the individualization of chemotherapeutic regimes. The quality of growth which would enable chemosensitivity to be assessed most precisely (Type II growth) is generally characteristic of patients in very advanced stages of disease, when such an assessment is of least value. Evidence of clonal heterogeneity has not only been found in this study, but has also been demonstrated by MacKintosh *et al.* (1981) who showed that 5 cell lines derived from individual agar clones grown from a single human ovarian tumour specimen, exhibited differing patterns of growth, morphology and drug sensitivity in culture.

In the light of these reservations and those of other workers (Rupniak & Hill, 1980; Selby & Raghavan, 1981) further basic research aimed at the optimization of this assay system is required. These results also highlight the need for a reliable animal model for the study of tumour progression and of the development of clonal heterogeneity within tumours.

This project was supported by the Peter Crimmins Research Fund. I. Bertoncello is a Peter Crimmins Research Flllow at the Cancer Institute. We would like to thank Dr P. Ironside and Mary Seyfang, Pathology Department of the Cancer Institute, for evaluating the biopsy specimens prior to culture. We are also indebted to Wendy Morgan of the Medical Records Unit of the Cancer Institute for her invaluable assistance in collating the relevant clinical data from patient histories. Our thanks also to Debbie Cruickshank of Compstat, Cancer Institute, for her assistance with statistical analysis of survival data.

### NOTE ADDED IN PROOF

Our attention has been drawn to a recent publication supporting our findings that significant clonal heterogeneity exists, both in initial cloning capacity and in self-renewal of primary clones. Buick, R. N. & Mackillop, W. J. (1981, Measurement of self-renewal in culture of clonogenic cells from human ovarian carcinoma. *Br. J. Cancer*, **44**, 394) have demonstrated a marked patient-to-patient variation in relf-renewal capacity of clonogenic cells, and that not all primary clonogenic cells could undergo self-renewal during clonal suspension in culture.

### REFERENCES

Alberts, D. S., Salmon, S. E., Chen, H. S. G. & 4 others (1980) *In vitro* clonogenic assay for predicting response of ovarian cancer to chemotherapy. *Lancet*, ii, 340.

Barrett, J. C. & Ts'o, P. O. P. (1978) Evidence for the progressive nature of neoplastic transformation *in vitro*. *Proc. Natl Acad. Sci.*, **75**, 3761.

BRADLEY, T. R., HODGSON, G. S. & ROSENDAAL, M. (1978) The effect of oxygen tension on haemopoietic and fibroblast cell proliferation *in vitro*. *J. Cell Physiol.*, **94**, 517.

BUICK, R. N., FRY, S. E. & SALMON, S. E. (1980) Effect of host-cell interactions on clonogenic carcinoma cells in human malignant effusions. *Br. J. Cancer*, **41**, 695.

CARNEY, D. N., GAZDAR, A. F. & MINNA, J. D. (1980) Positive correlation between histological tumor involvement and generation of tumor cell colonies in agarose in specimens taken directly from patients with small cell carcinoma of the lung. *Cancer Res.*, **40**, 1820.

COURTENAY, V. D. & MILLS, J. (1978) An *in vitro* colony assay for human tumours grown in immune-suppressed mice and treated *in vivo* with cytotoxic agents. *Br. J. Cancer*, **37**, 261.

COURTENAY, V. D., SELBY, P. J., SMITH, I. E., MILLS, J. & PECKHAM, M. J. (1978) Growth of human tumour cell colonies from biopsies using two soft-agar techniques. *Br. J. Cancer*, **38**, 77.

DANES, B. S. (1980) Heritable colonic cancer syndromes: Induction of anchorage-independence in dermal cultures derived from patients with adenomatosis of the colon and rectum. *Oncology*, **37**, 386.

HAMBURGER, A. W. & SALMON, S. E. (1977) Primary bioassay of human tumour stem cells. *Science*, **197**, 461.

HAMBURGER, A. W., SALMON, S. E., KIM, M. B. & 4 others (1978) Direct cloning of human ovarian carcinoma cells in agar. *Cancer Res.*, **38**, 3438.

HARD, G. C., KING, H., BORLAND, R., STEWART, B. W. & DOBROSTANSKI, B. (1977) Length of *in vivo* exposure to a carcinogenic dose of dimethylnitrosamine necessary for subsequent expression of morphological transformation by rat kidney cells *in vitro*. *Oncology*, **34**, 16.

IOACHIM, H. L., SABBATH, M. ANDERSSON, B. & BARBER, H. R. K. (1974) Tissue cultures of ovarian carcinomas. *Lab. Invest.*, **31**, 381.

LABOISSE, C. L., AUGERON, C. & POTET, F. (1981) Growth and differentiation of human gastrointestinal adenocarcinoma stem cells in soft agarose. *Cancer Res.*, **41**, 310.

MACKINTOSH, M. D., LOUIE, A. C. & EVANS, T. L. (1981) Clonal heterogeneity in a human ovarian adenocarcinoma. *Proc. 72nd AACR and 17th ASCO.*, **22**, 379.

MIHICH, E., LAURENCE, D. J. R., LAWRENCE, D. M. & ECKHARDT, S. (1979) UICC Workshop in human tumor sampling for biochemical pharmacological studies of target determinants of drug action. *UICC Tech. Rep. Series*, **43.**

MOURIQUAND, J., MOURIQUAND, C., PETITPAS, E. & MERMET, M. A. (1978) Long-term tissue cultures of human pleural effusions: A cytological follow-up. *In Vitro*, **14**, 591.

NEUGUT, A. I. & WEINSTEIN, B. (1979) The use of agarose in the determination of anchorage-independent growth. *In Vitro*, **15**, 351.

NOWELL, P. C. (1976) The clonal evolution of tumor cell populations. *Science*, **194**, 23.

PAVELIC, Z. P., SLOCUM, H. K., RUSTUM, Y. M. & 5 others (1980) Growth of cell colonies in soft agar from biopsies of different human solid tumors. *Cancer Res.*, **40**, 4151.

PAVELIC, Z. P., SLOCUM, H. K., RUSTUM, Y. M., POLYANSKAYA, C., KARAKOURIS, H. T. & MITTELMAN, A. (1981) Insights on biology of human solid tumor cloning *in vitro*. *Proc. 72nd AACR and 17th ASCO.*, **22**, 387.

PUCK, T. T. & MARCUS, P. I. (1956) Action of X-rays on mammalian cells. *J. Exp. Med.*, **103**, 653.

ROSCOE, J. P., HINCE, T. A., CLAISSE, P. J. & WINSLOW, D. P. (1980) Effect of 12-O-tetradecanoylphorbol-13-acetate on two characteristics of transformation acquired sequentially by ENU-exposed rat brain cells. *Br. J. Cancer*, **42**, 756.

RUPNIAK, H. T. & HILL, B. T. (1980) The poor cloning ability in agar of human tumour cells from biopsies of primary tumours. *Cell Biol. Int. Reps.* **4**, 479.

SALMON, S. E., HAMBURGER, A. W., SOEHNLEN, B., DURIE, B. G. M., ALBERTS, D. S. & MOON T. E. (1978) Quantitation of differential sensitivity of human tumor stem cells to anticancer drugs. *N. Engl. J. Med.*, **298**, 1321.

SELBY, P. J. & RAGHAVAN, D. (1981) Role of laboratory chemosensitivity testing in the selection of cancer chemotherapy for individual patients. *J. Clin. Pathol.*, **34**, 455.

SHIN, S., FREEDMAN, V. H., RISSER, R. & POLLACK, R. (1975) Tumorigenicity of virus-transformed cells in nude mice is correlated specifically with anchorage-independent growth. *Proc. Natl Acad. Sci.*, **72**, 4435.

TOLMACH, L. J. & MARCUS, P. I. (1960) Development of X-ray induced giant HeLa cells. *Exp. Cell Res.*, **20**, 350.

TUBIANA, M. & MALAISE, E. P. (1976) Growth rate and cell kinetics in human tumours: Some prognostic and therapeutic implications. In *Scientific Foundations of Oncloogy*, (Eds. Symington & Carter). London: Heinemann p. 126.

VON HOFF, D., CASPER, J., BRADLEY, E. & 5 others (1980) Direct cloning of human neuroblastoma cells in soft-agar culture. *Cancer Res.*, **40**, 3591.